# Distribution and engagement of specialist doctors in public hospitals in Indonesia

**DOI:** 10.1186/1471-2458-14-S1-O15

**Published:** 2014-01-29

**Authors:** Andreasta Meliala

**Affiliations:** 1 Faculty of Medicine, Universitas Gadjah Mada (UGM), Yogyakarta 55281, Indonesia

## Background

Unequal distribution of specialist doctors in Indonesia has been identified many years ago. Government has taken several measures to address this issue. However the phenomenon is still present today. In the year 2014, Indonesia will apply universal health coverage which needs specialist doctors to support it. The failure to provide specialist doctors would increase inequity, particularly for those who live in islands, border, and remote areas. On the other hand, specialist doctors who are appointed by public hospitals as full-time doctors, do a dual practice. They spend more time in other institutions rather than in public hospitals. This study aimed to examine the association among demographic factors, number of hospitals, type of hospitals with distribution of specialist doctors, and to measure the level of engagement of specialist doctors in the public hospitals as their main designation.

## Materials and methods

A mixed method was used, emphasising on quantitative approach, which supported by qualitative approach to explain the previous findings. Secondary data measuring the distribution of specialist doctors and related variables were collected from the MoH and National Bureau of Statistic. Primary data were collected from 3 public hospitals in 3 different areas, representing 3 different characteristics of distributions. Statistical and qualitative analyses were employed to examine the data. The Ethical Committee of Faculty of Medicine UGM enacted ethical clearance for this research.

## Results

Distribution of specialist doctors were positively associated with population, number of private hospitals and negatively associated with fiscal capacity and number of public hospitals. Distribution pattern in Indonesia has been identified as static distribution and dynamic distribution. In the static distribution areas, the existence of specialist doctors could not be found. Engagement of specialist doctors in the public hospitals was associated with professional facilities, medical fees, and flexible hours provided by the hospital managers. Barrier to entry was found as a negative factor to increase engagement level.

## Conclusions

Distribution of specialist doctors in Indonesia ranged from static distribution areas to dynamic distribution area. Within the dynamic distribution areas, engagement level of specialist doctors in the hospitals was low. It implied to the pattern of specialist doctor distribution in general. Both factors, type of distribution and engagement level, created a circle to affect distribution of specialist doctors in Indonesia. Policy makers should develop special policy to improve distribution of specialist doctors in particular area and to endorse new concept of “sustainability of services provided by simultaneous team”.

